# Comparative Analysis of Mesenchymal Stem Cell Cultivation in Fetal Calf Serum, Human Serum, and Platelet Lysate in 2D and 3D Systems

**DOI:** 10.3389/fbioe.2020.598389

**Published:** 2021-01-15

**Authors:** Marline Kirsch, Jessica Rach, Wiebke Handke, Axel Seltsam, Iliyana Pepelanova, Sarah Strauß, Peter Vogt, Thomas Scheper, Antonina Lavrentieva

**Affiliations:** ^1^Institute of Technical Chemistry, Leibniz University Hannover, Hanover, Germany; ^2^German Red Cross Blood Service NSTOB, Institute Springe, Springe, Germany; ^3^Bavarian Red Cross Blood Service, Institute Nuremberg, Nuremberg, Germany; ^4^Department of Plastic, Aesthetic, Hand and Reconstructive Surgery, Hannover Medical School, Hanover, Germany

**Keywords:** platelet lysate, mesenchymal stem cells, differentiation, medium supplements, fetal calf serum, human serum, gelatin methacryloyl (GelMA), hydrogel

## Abstract

*In vitro* two-dimensional (2D) and three-dimensional (3D) cultivation of mammalian cells requires supplementation with serum. Mesenchymal stem cells (MSCs) are widely used in clinical trials for bioregenerative medicine and in most cases, *in vitro* expansion and differentiation of these cells are required before application. Optimized expansion and differentiation protocols play a key role in the treatment outcome. 3D cell cultivation systems are more comparable to *in vivo* conditions and can provide both, more physiological MSC expansion and a better understanding of intercellular and cell-matrix interactions. Xeno-free cultivation conditions minimize risks of immune response after implantation. Human platelet lysate (hPL) appears to be a valuable alternative to widely used fetal calf serum (FCS) since no ethical issues are associated with its harvest, it contains a high concentration of growth factors and cytokines and it can be produced from expired platelet concentrate. In this study, we analyzed and compared proliferation, as well as osteogenic and chondrogenic differentiation of human adipose tissue-derived MSCs (hAD-MSC) using three different supplements: FCS, human serum (HS), and hPL in 2D. Furthermore, online monitoring of osteogenic differentiation under the influence of different supplements was performed in 2D. hPL-cultivated MSCs exhibited a higher proliferation and differentiation rate compared to HS- or FCS-cultivated cells. We demonstrated a fast and successful chondrogenic differentiation in the 2D system with the addition of hPL. Additionally, FCS, HS, and hPL were used to formulate Gelatin-methacryloyl (GelMA) hydrogels in order to evaluate the influence of the different supplements on the cell spreading and proliferation of cells growing in 3D culture. In addition, the hydrogel constructs were cultivated in media supplemented with three different supplements. In comparison to FCS and HS, the addition of hPL to GelMA hydrogels during the encapsulation of hAD-MSCs resulted in enhanced cell spreading and proliferation. This effect was promoted even further by cultivating the hydrogel constructs in hPL-supplemented media.

## Introduction

More than half a century has passed since the first isolation and *in vitro* cultivation of mesenchymal stem cells (MSC) described by Friedenstein et al. (1966) and Friedenstein et al. (1970). From that point on, numerous studies about the handling of these cells have been performed. However, the development of an optimal protocol for cultivating MSCs is still in progress (Spees et al., 2016; Lavrentieva et al., 2020). The most common and most widely used cell culture medium supplement is fetal calf serum (FCS). It has been used for the cultivation of several cell types for more than 50 years (Gstraunthaler et al., 2013). To date, most cell isolation or expansion protocols for clinical studies use FCS for supplementation (Schrödel, 2007; Lindroos et al., 2009; Bieback, 2013; Hemeda et al., 2014; Burnouf et al., 2016; Monsanto et al., 2017; Motedayyen et al., 2017; Araújo et al., 2018; Lee et al., 2019; Cherian et al., 2020; Ghamari et al., 2020; Wagner et al., 2020). Even though there are various disadvantages related to FCS such as lot-to-lot variability, ethical concerns about collecting the serum from the heart of unborn calves and the risk of viral, mycoplasm or prion infections or immune responses of the recipients toward foreign factors, there are no widely accepted alternatives for FCS (Lindroos et al., 2009; Bieback, 2013; Jonsdottir-Buch et al., 2013; Hemeda et al., 2014; Burnouf et al., 2016; Monsanto et al., 2017; Motedayyen et al., 2017; Lee et al., 2019; Cherian et al., 2020; Wagner et al., 2020).

A sustained effect on the differentiation capacity and the immunophenotype of cells has been observed in different studies by using xeno-free autologous human serum (HS) as a medium supplement. Due to its promoting effect on cell expansion and its human origin, HS appears to be a potential alternative to FCS (Mannello and Tonti, 2007). However, because of high costs of its manufacture, the production of HS is actually decreasing (Müller et al., 2006; Mannello and Tonti, 2007; Aldahmash et al., 2011; Hemeda et al., 2014). Hence, the goal for the future is a completely chemically defined MSC medium. But to date, there is still no reliable, efficient, comprehensive and fully defined medium available for a broad MSCs cultivation. Moreover, most of the available defined media require additional coatings of the cell culture surface with proteins (Pijuan-Galitó et al., 2016; Salzig et al., 2016; Wu et al., 2016; Cherian et al., 2020). These, in turn are often derived from animal origin, so that in this case no truly xeno-free cultivation with these media is possible.

MSCs have a clinical potential for use in cell therapies and tissue engineering (TE) due to their immunomodulatory potential, stromal functions and their great proliferation as well as differentiation capacities *in vitro* (Dominici et al., 2006). MSCs can be differentiated in different cell types and the potential of controlled chondrogenic and osteogenic differentiation of these cells makes them promising candidates for cartilage and bone TE, as well as for *in vitro* 2D and 3D models for drug screening and disease modeling (Raic et al., 2019). Because of rising ethical, safety and scientific concerns, the World Health Organization and Good Manufacturing Practice (GMP) guidelines recommend the prohibition of the use of animal-derived supplements or supplements containing animal-sourced ingredients for stem cell cultivations or advanced therapy medicinal products (Schrödel, 2007; Lindroos et al., 2009; Bieback, 2013; Gstraunthaler et al., 2013; Hemeda et al., 2014). Hence, until a chemically defined medium is available for MSCs cultivation, it is essential to evaluate existing alternatives to FCS such as human platelet lysate (hPL).

The first attempts to use platelet-rich plasma and platelet lysates as cell culture medium supplement were already made 30 years ago (Gimbrone et al., 1969; Mavrina et al., 1986; Burnouf et al., 2016). Since that time it has been shown that hPL supports *in vitro* growth and osteogenic differentiation of MSCs (Doucet et al., 2005; Lange et al., 2007; Schallmoser et al., 2007; Bieback et al., 2009; Jonsdottir-Buch et al., 2013; Schallmoser and Strunk, 2013; Shih and Burnouf, 2015; Siciliano et al., 2015; Astori et al., 2016; Burnouf et al., 2016; Fernandez-Rebollo et al., 2017), as well as the proliferation of progenitor cells and endothelial colony forming progenitor cells (Schallmoser and Strunk, 2013). hPL appears to be a valuable alternative to FCS and shows several advantages, such as its easy production from human platelet concentrate in conformity with GMP guidelines (Burnouf et al., 2016). Moreover, the use of hPL combines modern social principles of ethics, sustainability, recycling and resources conservation. More than twenty percent of the platelets donated in global blood donation programs expire before they can be used for infusions. Since no difference was determined between using expired or fresh hPL as medium supplements, there is a possibility to recycle expired platelets obtained from blood banks (Jonsdottir-Buch et al., 2013). Furthermore, hPL contains many bioactive factors such as growth factors and cytokines, which act synergistically to support the cell growth, behavior and differentiation of MSCs. Moreover, hPL has demonstrated the ability to enhance the proliferation and differentiation of MSCs in 2D and 3D cultivations in various studies (Supplementary Table 1) (Bieback, 2013; Jonsdottir-Buch et al., 2013; Altaie et al., 2016; Burnouf et al., 2016; Kirsch et al., 2019, 2020). However, commercially available hPLs usually contain heparin in order to prevent hPL gelation. Heparin is a product, extracted from porcine small intestine mucosa. Thus, cell cultures grown in the presence of such hPL are no longer xeno-free (Mojica-Henshaw et al., 2013). Furthermore, Hemeda et al. showed that the heparin concentration is critical for 2D MSC cultures in hPL-supplemented medium. This group demonstrated a concentration-dependent influence of heparin on cell proliferation, the colony-forming unit frequency as well as the *in vitro* differentiation of MSCs (Hemeda et al., 2013). In our study, fibrinogen-depleted hPL was used without addition of heparin to the cell culture medium, in order to maintain true xeno-free cultivation conditions. Providing genuine xeno-free conditions and considering these promising aspects, the challenge is to evaluate and optimize protocols for the production and application of hPL.

Many cell cultivation protocols are still predominantly designed for 2D cultivation. However, compared to 2D-cultured MSCs, 3D cell cultivation seems to be more advantageous, because 3D growing cells reflect the *in vivo* environment of MSCs to a higher extent. Thus, physiological cell-cell and cell-matrix contacts can be simulated and studied only in 3D cell cultures. Moreover, considering angiogenic and immunomodulatory factors, 3D-grown cells have a higher quality (Mark et al., 1977; Cukierman et al., 2001, 2002; Abbott, 2003; Bissell et al., 2003; Schmeichel and Bissell, 2003; Lee et al., 2009; Li and Cui, 2014). Various studies have already investigated the possibility of direct 3D isolation of MSCs from bone and adipogenic tissue to prevent 2D cultivation of the cells (Papadimitropoulos et al., 2014; Egger et al., 2019). Similar to 2D cell cultivation, xeno-free alternatives to FCS are needed for 3D cell cultivation and the influence of medium supplements must be systematically evaluated.

In addition to cellular aggregates and cells growing on scaffolds, hydrogels represent a very promising 3D cultivation system. Hydrogels provide a tunable versatile platform for *in vitro* 3D cultivation, TE and bioprinting (Ruedinger et al., 2015; Pepelanova et al., 2018). Regular semi-synthetic hydrogels are usually stored as lyophilized proteins and are reconstituted with PBS prior cell encapsulation. Thus, they do not normally contain any additional supplements. Regarding 3D hydrogels, the addition of supplements to both, hydrogels and the medium can influence cell behavior. Only a limited number of studies have investigated the influence of direct hPL addition to hydrogels on encapsulated MSCs (Moreira Teixeira et al., 2012; Santos et al., 2018; Jooybar et al., 2019). Most of these studies cultivated the hPL-containing hydrogels in media supplemented with FCS, thus effectively not under true xeno-free conditions (Moreira Teixeira et al., 2012; Santos et al., 2018). So far, no study has shown the influence of different medium supplements on the cell behavior of encapsulated MSCs in comparison. To the best of our knowledge, no study investigating the effect of supplementation of hydrogels and medium on the encapsulated cells has been conducted to date.

In the present study, the influence of hPL (2.5% hPL) on the proliferation, as well as the osteogenic and chondrogenic differentiation of human adipose-derived mesenchymal stem cells (hAD-MSCs) obtained from four different donors was systematically investigated in a 2D cultivation system.

The influence of hPL was compared to FCS (10%) and HS (10%). Online monitoring of osteogenic differentiation in 2D under the influence of different supplements was performed and evaluated.

Recently, we published a study about the influence of formulating GelMA hydrogels with different hPL concentrations (Kirsch et al., 2019). The addition of hPL directly to the hydrogel supported not only the cells but also had a positive impact on the mechanical properties of the GelMA hydrogels. It was demonstrated that the addition of hPL to the hydrogels improves cell growth and cell adhesion. However, this beneficial effect could have been caused by the direct formulation of the hydrogel with a supplement carrying multiple bioactive factors, and must not be directly related to the superior properties of a xeno-free protocol with hPL. In order to investigate this aspect more closely, we expanded the study by formulating GelMA hydrogel with three different media supplements (FCS, HS, and hPL) and studied their influence on cell growth and adhesion. Consequently, the influence of the three supplements as direct additions to the growth media of 3D cultivated cells was also investigated under the aspects of cell spreading, cell morphology, as well as cell viability.

## Materials and Methods

### MSC Cultivation

Human AD-MSCs were isolated from adipose tissue of four donors after abdominoplasty surgery. The use of human tissue from patients (after their informed consent) has been approved by the Institutional Review Board (Hannover Medical School, Ref. Nr.: 3475-2017). As described earlier, we performed surface marker analysis and functional characterization of the isolated cell populations to characterize them as MSCs (Pepelanova et al., 2018). hAD-MSCs were expanded in alpha-MEM medium (Thermo Fisher Scientific, Waltham, MA, USA) and 10% human serum (CC-pro, Oberdorla, Germany) as well as 0.5% gentamicin (Merck Millipore, Darmstadt, Germany), harvested by accutase treatment (Sigma Aldrich, Taufkirchen, Germany), and cryopreserved at passage one or two until the start of the experiment. Experiments were performed with cells of passages two to nine. Following concentrations of cell culture supplements were used for all performed experiments: 10% FCS, 10% HS and 2.5% hPL. hPL concentration of 2.5% was chosen based on preliminary experiments. The tested concentrations in preliminary experiments were 0, 1, 2.5, 5, and 10% of hPL in medium. The highest differentiation capacity and a more even distribution of Alizarin Red staining were observed at a concentration of 2.5% (Supplementary Figure 1a). Furthermore, in order to test if lower concentrated FCS or HS could also result in higher osteogenic differentiation, 2.5% of all supplements were used in preliminary experiments. The cell viability and osteogenic differentiation was significantly lower for hAD-MSCs cultivated with 2.5% FCS and 2.5% HS compared to 2.5% hPL (Supplementary Figure 1b). Due to those results the standard concentration of 10% FCS and HS was used to supplement the medium in all experiments.

### Platelet Lysate Preparation

Human platelet lysates were prepared and provided by the German Red Cross Blood Service NSTOB (Springe, Germany) by freeze-thaw treatments of pooled platelets from surplus buffy coats. To prevent gelation of hPL and avoid the use of heparin, fribrinogen depletion was performed by the calcium presipitation method, followed by filtration of the platelet lysate.

### Cell Proliferation Analysis

For cell proliferation studies, the hAD-MSCs were harvested at 80% of confluency by accutase treatment, counted using a hemocytometer and seeded at a density of 3,000 cells/cm^2^ in 25 cm^2^ cell culture flasks containing 4 ml medium. Alpha-MEM medium was supplemented with 10% HS, 10% FCS or 2.5% hPL. hAD-MSCs from four different donors were sub-cultivated two times per week with a seeding density of 3,000 cells/cm^2^ over five passages; cell number and viability were evaluated by trypan blue exclusion and cumulative cell numbers were calculated.

### Osteogenic and Chondrogenic Differentiation

For differentiation experiments, cells were seeded in 24-well plates (growth area 2 cm^2^, Sarstedt, Germany) at a density of 10.000 to 15.000 cells/cm^2^ in 500 μl alpha-MEM containing 10% HS, 10% FCS or 2.5% hPL and 50 μg/ml gentamicin per well. After seeding, cells were cultivated for 24–48 h (until full confluence was achieved) in a humidified atmosphere containing 5% CO_2_ and 21% O_2_ at 37°C. Afterwards, the cell culture medium was changed to either the osteogenic or chondrogenic differentiation medium. Osteogenic differentiation medium contained 5 mM β-glycerophosphate, 0.1 μM dexamethasone, 0.2 mM L-ascorbate-2-phosphate, 0.5% gentamicin, as well as 2.5% hPL, 10% HS or 10% FCS. Serum-free chondrogenic differentiation medium was purchased from Gibco (StemPro Chondrogenic differentiation kit, Gibco, Germany) and also supplemented with 2.5% hPL, 10% HS or 10% FCS. The medium was exchanged every 3–4 days. Cells were cultured for the next 7, 14, or 21 days and then washed in PBS and fixed for 15 min at 4°C with 4% paraformaldehyde for staining. Before fixation, indirect cell viability was estimated by CellTiter-Blue^®^ (CTB) assay according to the manufacturer's instructions (Promega, Mannheim, Germany). Briefly, CTB stock solution was diluted in alpha-MEM basal medium (1:10 v/v) and added to the cells. The fluorescence was measured after 2 h of incubation at an extinction wavelength of 544 nm and an emission wavelength of 590 nm with a microplate reader (Fluoroskan Ascent, Thermo Fisher Scientific Inc., Waltham, MA, USA).

### Alizarin Red and Alcian Blue Staining

In order to determine the degree of osteogenic differentiation, Alizarin Red staining was used. The fixed cell layers were incubated in Alizarin Red solution, containing 1% Alizarin Red S (Merck KGaA, Darmstadt, Germany) in deionized H_2_O, for 15 min at room temperature. After washing with deionized H_2_O the red chelates were detected with a microscope. The accumulation of proteoglycans in the extracellular matrix during the chondrogenic differentiation was visualized by using Alcian Blue staining. Fixed cell layers were washed twice with PBS, incubated for 3 min in 3% acetic acid at room temperature, followed by 30 min incubation in Alcian Blue solution (1% Alcian Blue 8GX, Sigma Aldrich, in 3% acetic acid) at room temperature. After incubation, cell layers were washed several times with 3% acetic acid and the presence of bound Alcian Blue stain was detected using a microscope.

### Alizarin Red Extraction

To quantify the degree of osteogenic differentiation, Alizarin Red was extracted with 10% hexadecylpyridinium chloride monohydrate (Sigma-Aldrich, St. Louis, WI, USA) in 1 × PBS for 20 min at 37°C. The concentration of extracted Alizarin Red was measured at 550 nm (Epoch, BioTek Instruments, Winooski, VT, USA) and calculated using a calibration curve with a regression of 0.997. If required, samples were diluted to bring Alizarin Red concentrations within the linear range of the photometer.

### Online Monitoring and Evaluation of Osteogenic Differentiation by Image Analysis

During the differentiation, time-lapse microscopic pictures were taken using an IncuCyte^®^ Live-Cell Imaging System (Sartorius, Göttingen, Germany) placed in the incubator. The osteogenic differentiation of the cells was determined and quantified by training a metric phase object confluence mask for the typical changes in the morphology of the cells toward osteocytes (Figure 5B). To add an enhanced coloring mask to the images, the software (IncuCyte^®^ Analysis Software) provides a processing definition step to train the algorithm to highlight the correct markers on a limited representative set of images at different time points and stages of the osteogenic differentiation. This processing step can be visually inspected and adjusted to be valid for all the pictures in the limited training set. After the visual inspection, when the mask highlights the correct parts where the cells are slowly changing their morphology toward osteocytes and increasing the mineralization of the extracellular matrix, the software can automatically analyze the images of the total experiment. When the processing definition is valid for the training image set, this can be used for online monitoring of the experiment.

### Alkaline Phosphatase (ALP) Assays

Next to the Alizarin Red staining and quantification, the ALP activity was also measured in the cells as well as in the supernatants (Anh et al., 1998). The fixated cells were incubated with 5-bromo-4-chloro-3-indolyl phosphate (BCIP)/nitro blue tetrazolium (NBT) (SIGMAFAST BCIP^®^/NBT, B5655, Merck, Darmstadt, Germany) for 30 min at room temperature, washed with PBS and microscopically analyzed with an Olympus IX50 (Olympus Corporation, Tokyo, Japan). For the quantification of the ALP activity in the supernatant 1 day before measurement, the medium was exchanged to proliferation or differentiation medium without supplements, in order to ensure a sensitive measurement of the activity in the medium. As a standard 4-nitrophenol solution (10 mM, Sigma-Aldrich, St. Louis, WI, USA) was used and diluted in ALP buffer (one Trizma® Buffer tablet of SIGMAFAST™ p-Nitrophenyl phosphate dissolved in 20 ml ddH_2_O, Merck, Darmstadt, Germany) for a standard series. The five-fold concentrated substrate solution was prepared by dissolving one pNPP tablet and one Trizma® Buffer tablet of the kit in 4 ml ddH_2_O. Each week, the supernatant was removed 5 min centrifuged at 4°C/14.000 rpm and 250 μl were stored at −20°C until further use. At the end of the experiment, all supernatants were thawed, vortexed and 40 μl/well of each sample, control (medium) and the standard series was added in a 96-wellplate. After adding 10 μl of the substrate solution, the plate was shaked for 5 min at 37°C/450 rpm and incubated for 7 h at 37°C. After 7 h, the plate was measured at 405 nm (Epoch, BioTek Instruments, Winooski, VT, USA) and the ALP activity was calculated by using a calibration curve of the standard series with a regression of 0.999.

### Quantitative Glycosaminoglycan (GAG) Assay

After 7, 14, and 21 days of culture in chondrogenic differentiation medium, the cells were harvested and digested in 500 μl/well papain solution [500 μl 0.1 M NaH2PO4/0.005 mM EDTA (pH 6), 5 μl β-mercaptoethanol, and 2.5 μl papain (10 mg/ml, Sigma-Aldrich, St. Louis, WI, USA) at 60°C and 800 rpm overnight. The papain digest solution was then used to quantify the deoxyribonucleic acid (DNA) and GAGs. A DNA standard series was prepared with DNA from calf thymus (Sigma-Aldrich, St. Louis, WI, USA). In a 96 well plate, 100 μl preparation buffer and then 100 μl of the respective sample or standard series were added. After 100 μl of bisbenzimide (Sigma-Aldrich, St. Louis, WI, USA) was added to each well, the plate was measured at 360/460 nm with a spectrophotometer (F-7000 FL Spectrophotometer, Hitachi, Tokio, Japan). Chondroitin sulfate (Sigma-Aldrich, St. Louis, WI, USA) was used as a standard and 100 μl of the samples was added to 100 μl/well ddH_2_O in a 96 well plate. After adding 150 μl 1,9-dimethyl-methylene blue (DMMB, Sigma-Aldrich, St. Louis, WI, USA) the plate was measured at 530 nm (Epoch, BioTek Instruments, Winooski, VT, USA) and the DNA and GAG concentration was calculated with calibration curves.

### GelMA Synthesis and Hydrogel Preparation

**Figure 1:**
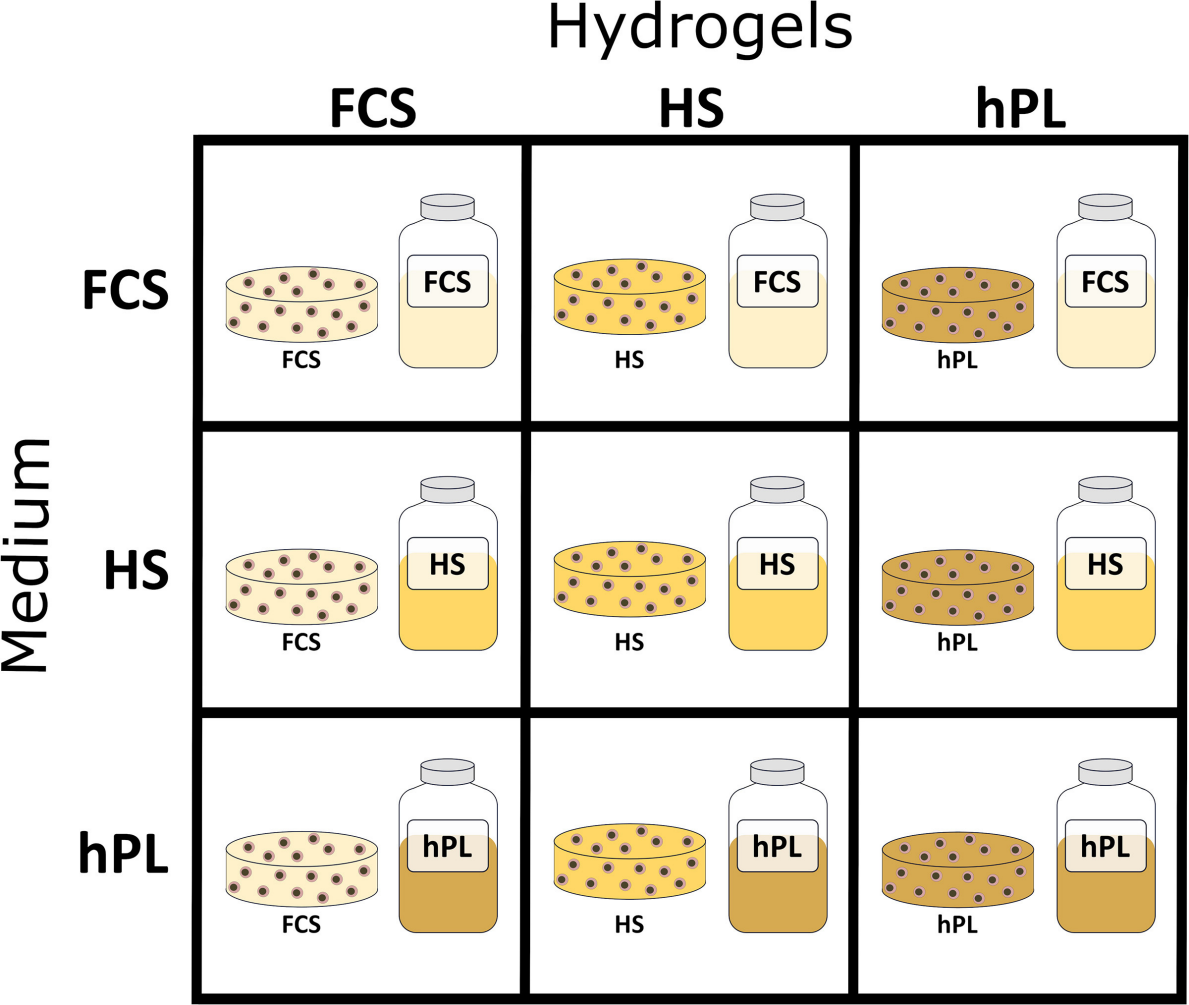
Schematic representation of 3D hydrogel-based cultivation: first GelMA hydrogels were formulated with 50% FCS, 50% HS, or 50% hPL followed by cultivation in medium supplemented with 10% FCS, 10% HS, or 2.5% hPL.

As already described in a previous study, GelMA was synthesized according to a previously described protocol (Pepelanova et al., 2018). The degree of functionalization (DoF) of GelMA used in the experiments was of 50%. GelMA solutions were prepared at a concentration of 5% (w/v). The GelMA was dissolved in 50% of PBS and either 50% (v/v) FCS, HS, or hPL (hPL pH value 7.3, manufactured by German Red Cross, Blood Service NSTOB, Springe, Germany) was added (Figure 1). After dissolving all GelMA solutions in a water bath at 37°C, they were sterile filtered with 0.45 μm polyethersulfone (PES) filters and 0.1% (w/v) photoinitiator 2-Hydroxy-4′-(2-hydroxyethoxy)-2-methylpropiophenone (Irgacure 2959) was added prior to the encapsulation of cells.

### Encapsulation and Cultivation of hAD-MSCs in Hydrogels

The cells were resuspended in the GelMA solutions at a concentration of 1.0 × 10^6^ cells/mL and filled in 50-μL disks (6-mm diameter) *in silicon* molds. With an UV intensity of 1.2 J/cm^2^ (polymerization time of ~5 min) the hAD-MSCs were encapsulated in the hydrogels with the help of a cross linker (BLX-365 Bio-Link, 365 nm, Vilber Lourmat, Germany). The cells encapsulated in GelMA hydrogels formulated with PBS, FCS, HS, and hPL were further cultivated in medium supplemented with 10% FCS, 10% HS, or 2.5% hPL (Figure 1).

The preparation of the well-plates and the handling of the hydrogels is described earlier (Kirsch et al., 2019). As described in more detail in our previous study, the indirect cell viability was determined by the CellTiter-Blue^®^ (CTB) (Promega, Mannheim, Germany) assay according to the manufacturer's specifications (Kirsch et al., 2019). For morphological analysis, encapsulated cells were cultivated for one, three or seven days, incubated in basal alpha-MEM with the addition of 4 μM calcein-acetoxymethyl (AM) (Merck, Darmstadt, Germany) for 40 min at 37°C. The hAD-MSCs were analyzed with a Cytation 5-Cell Imaging Multi-Mode Reader (Biotek Instruments, Winooski, VT, USA).

### Statistical Analysis

The data are presented as mean value ± standard deviation of the multiple measurements/counts of each sample. A one-way ANOVA (OriginLab) was performed to determine the statistical significance of the measured values, defined as *p*-value of ^*^p < 0.05, ^**^p < 0.01, or ^***^p < 0.001.

## Results

### hAD-MSCs Proliferation in 2D

**Figure 2:**
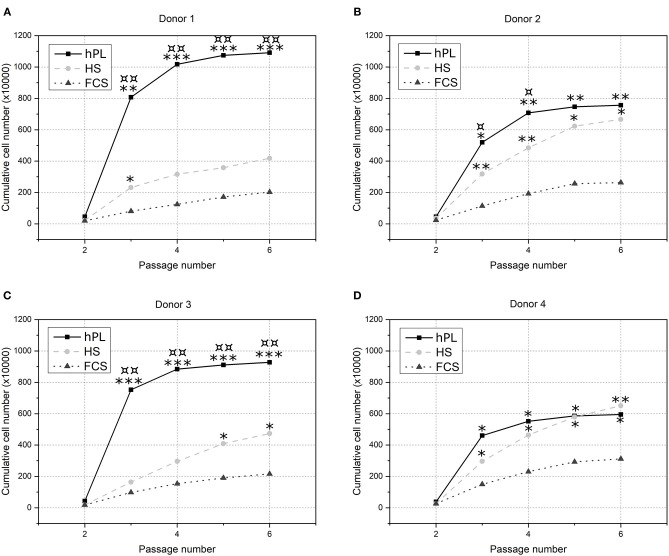
The influence of cell culture supplements on the long-term cultivation of hAD-MSCs. Cells from four donors [**(A)** Donor 1, **(B)** Donor 2, **(C)** Donor 3 and **(D)** Donor 4] were cultivated over five passages in 2.5% hPL (black line), 10% HS (light gray dotted line) or 10% FCS (gray dashed line) and cumulative cell numbers were calculated. *p < 0.05, **p < 0.01, ***p < 0.001 (*, **, *** indicates significant difference to FCS); ^¤^*p* < 0.05, ^¤¤^*p* < 0.01 (¤, ¤¤ indicates significant difference to HS).

To evaluate the influence of hPL on hAD-MSCs proliferation, cells isolated from four different donors were cultivated over five passages in alpha-MEM supplemented with 2.5% hPL and compared to the cell growth in conventional cell culture conditions: medium with 10% FCS and 10% HS. Differences in the cell growth of the various donors are caused by biological variations. Figures 2A–D, that three of the four donor cells cultivated in medium supplemented with hPL show the highest total cell number compared to FCS and HS as supplements. Especially during the first passages, the increase of cell number was highest for cells cultivated in hPL-containing medium. Cells cultivated in medium supplemented with FCS exhibited the lowest cell number and cell division for all donors.

To ensure that hAD-MSCs had not changed their typical immunophenotype during the cultivation with different supplements, a flow cytometry analysis for characteristic MSC- markers was performed and revealed no changes in specific marker expression (Supplementary Table 2).

The obtained results demonstrate that medium supplemented with hPL provide a significant higher cell proliferation of hAD-MSCs for three of four donors compared to HS and in all four donors compared to FCS as a supplement. The cumulative cell number after cultivation with hPL was twice to more than five times higher than the cell number after cultivation with FCS. In a short period of time the cell number can be increased significantly, which underlines the potential of hPL-containing medium for rapid MSCs expansion.

### Osteogenic Differentiation

**Figure 3:**
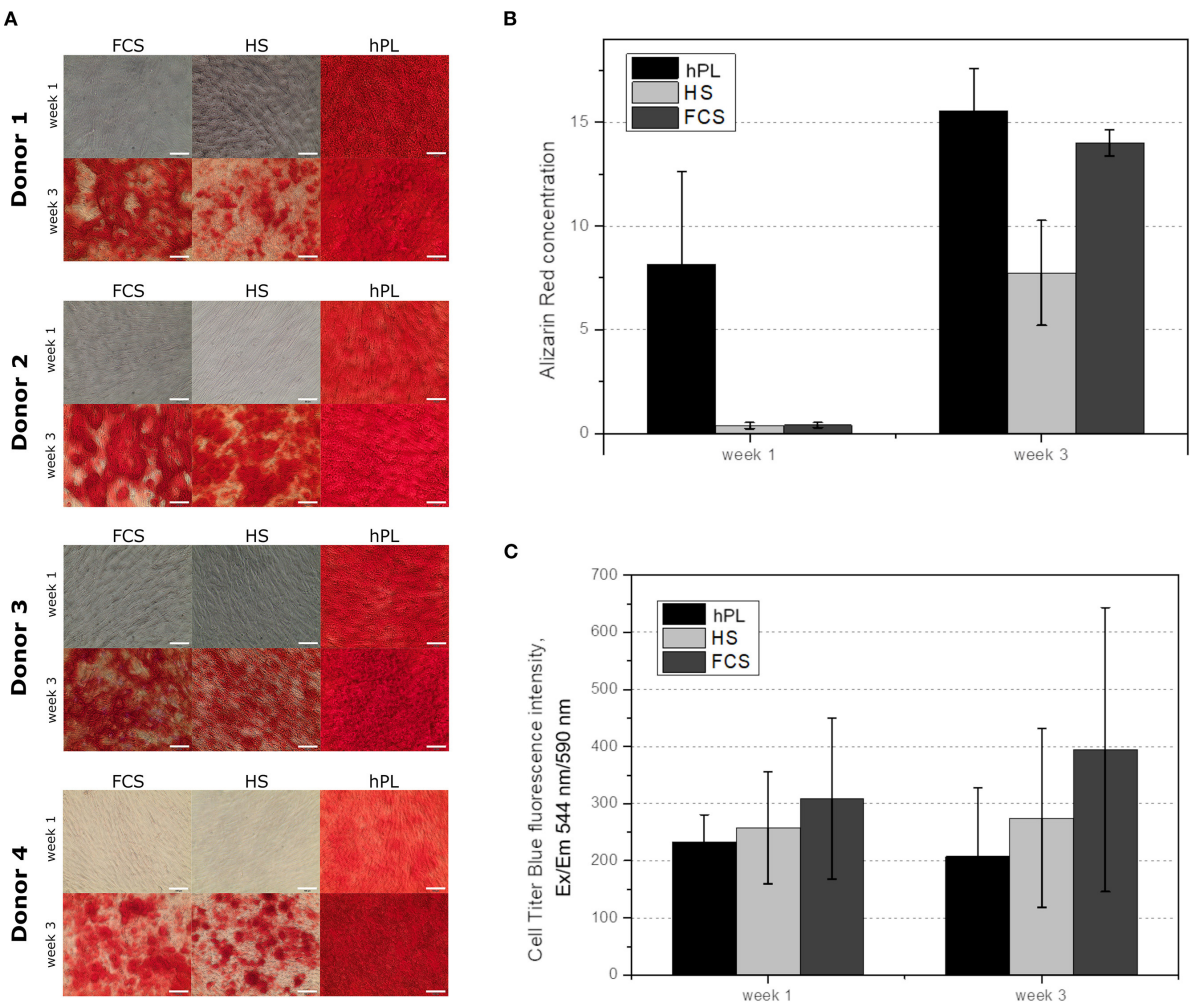
**(A)** Alizarin red staining of the hAD-MSCs from four donors differentiated in the presence of 2.5% hPL, 10% HS, or 10% FCS. The influence of cell culture supplements on the calcium deposition evaluated by Alizarin red extraction, 10× objective, scale bar 100 μm. **(B)** and on the cell viability during the differentiation **(C)**. Data represent the mean ± SD of a threefold determination for four donors.

**Figure 4:**
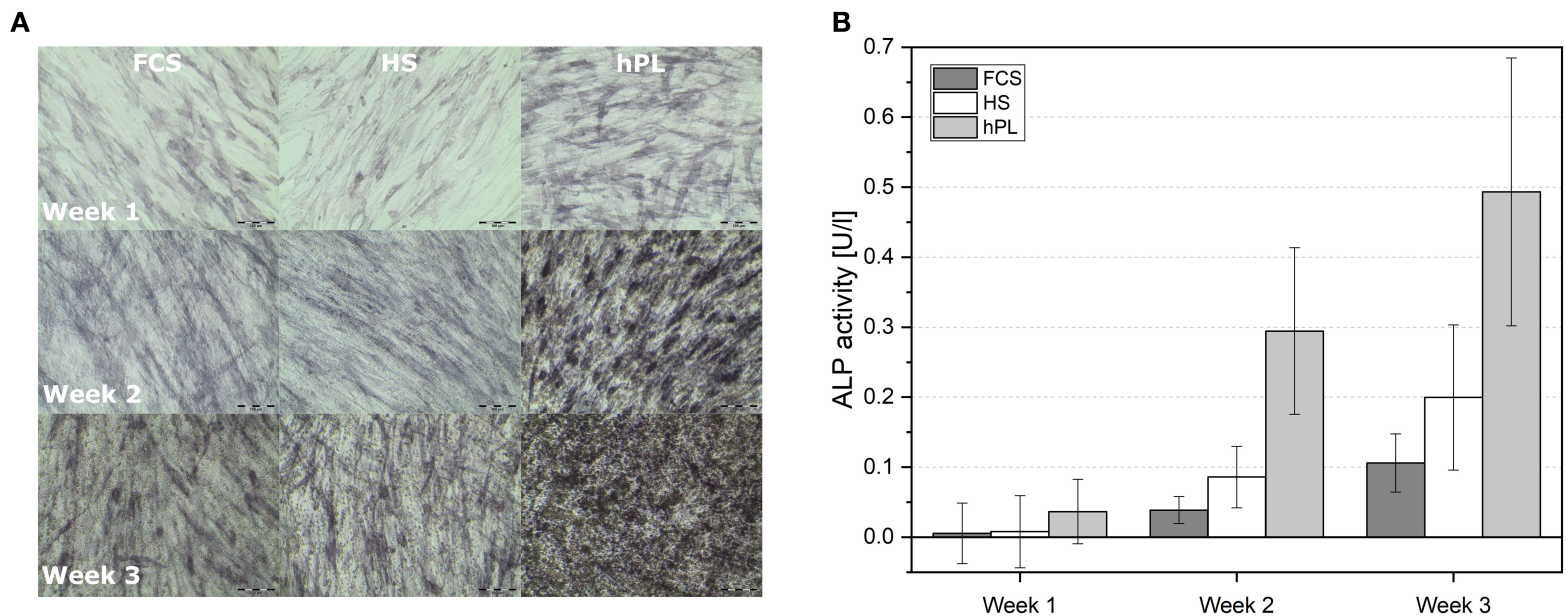
**(A)** Alkaline phosphatase staining of differentiated hAD-MSCs cultivated for 7, 14, and 21 days and differentiated in medium supplemented with FCS, HS and hPL, 10× objective, scale bar 100 μm. **(B)** Measured ALP activity in the supernatant of hAD-MSCs cultivated with FCS, HS and hPL for 7, 14, and 21 days. Data represent the mean ± SD of a threefold determination for two donors.

The cells were cultivated in differentiation medium supplemented with FCS, HS, or hPL over three weeks. Despite the higher amount of supplements (10% of volume), cells cultivated in medium with conventional supplements (FCS and HS) only showed an irregular Alizarin red staining after three weeks. As illustrated in Figure 3, only MSCs differentiated in medium supplemented with hPL showed an accumulation of calcium already after 1 week of cultivation visualized by Alizarin red staining. The staining was even more intense and homogenously distributed after 3 weeks of cultivation. hAD-MSCs from all donors exhibited an osteogenic differentiation in all used supplements after 3 weeks of stimulation. These results were also confirmed by the quantification of Alizarin red staining during the whole period of differentiation (Figure 3B). Only hPL as a supplement was able to induce osteogenic differentiation during the first week of cultivation. As illustrated in Figure 3C, the indirectly measured viability of cells remained high and did not change significantly over 3 weeks with all three medium supplements. In addition to the staining and quantification of Alizarin red, the ALP activity was measured in the supernatant and in the cells (Figure 4). Only cells differentiated with hPL containing medium showed ALP -positive cells already after 1 week (Figure 4A). In comparison, fewer ALP-positive cells were observed in FCS and HS differentiated cells. The quantitative determination of ALP activity in the supernatant showed the lowest activity each week in cells differentiated in FCS (Figure 4B). In HS differentiated cells, low ALP activity could be measured after seven days of differentiation. The highest ALP activity was detectable in the supernatant of cells differentiated in hPL-supplemented medium.

**Figure 5:**
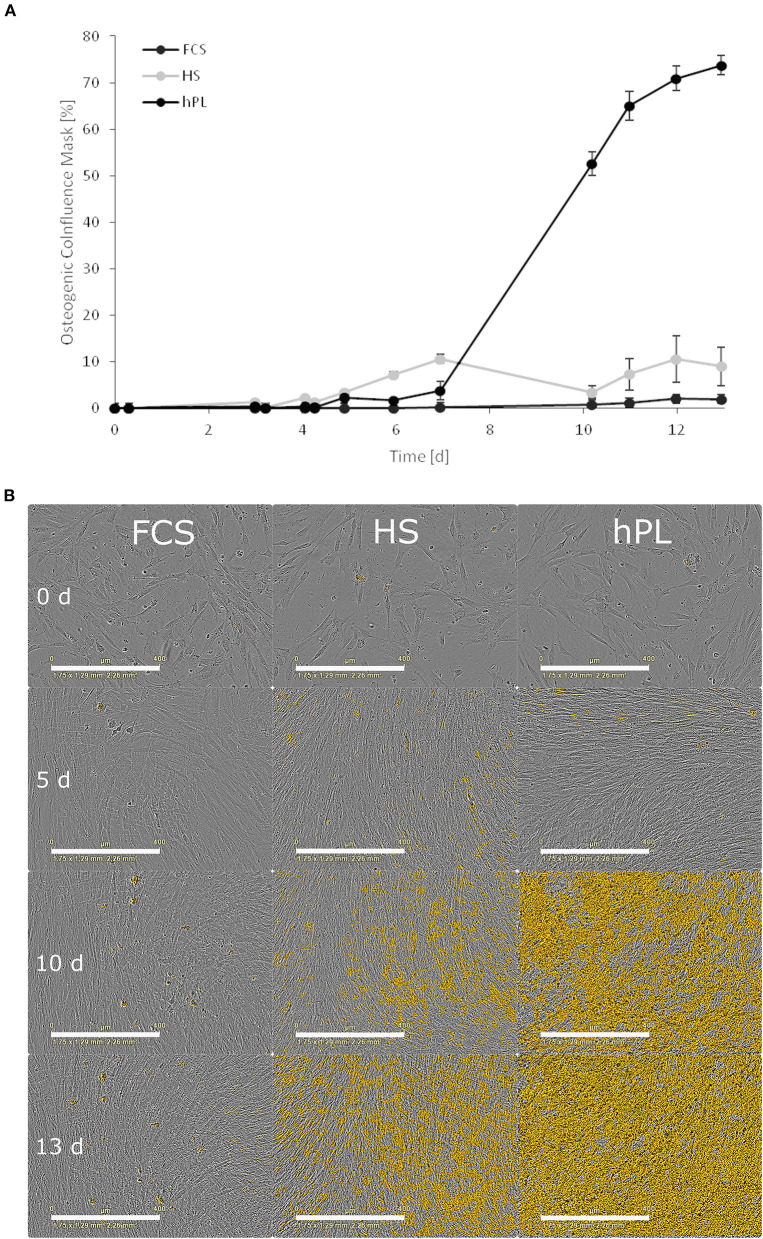
**(A)** The influence of cell culture supplements on the calcium deposition evaluated by using a trained metric phase object confluence mask for microscopic pictures analyzed by the IncuCyte Live-Cell Imaging System and Software (Essen BioScience, Ann Arbor, MI). Data represent the mean ± SD for a fourfold determination. **(B)** Microscopic pictures taken by an IncuCyte Live-Cell Imaging System and blended with a trained metric phase object confluence mask, which indicates typical osteogenic changes of the cells, scale bar 400 μm.

Online monitoring and the evaluation of osteogenic differentiation dynamics with the help of a trained metric phase object confluence mask also revealed the superiority of hPL in terms of the onset of calcium deposition (Figure 5). However, in comparison to the Alizarin red staining in the unstained live MSCs culture, the software first recognized the deposition after day seven.

### Chondrogenic Differentiation

**Figure 6:**
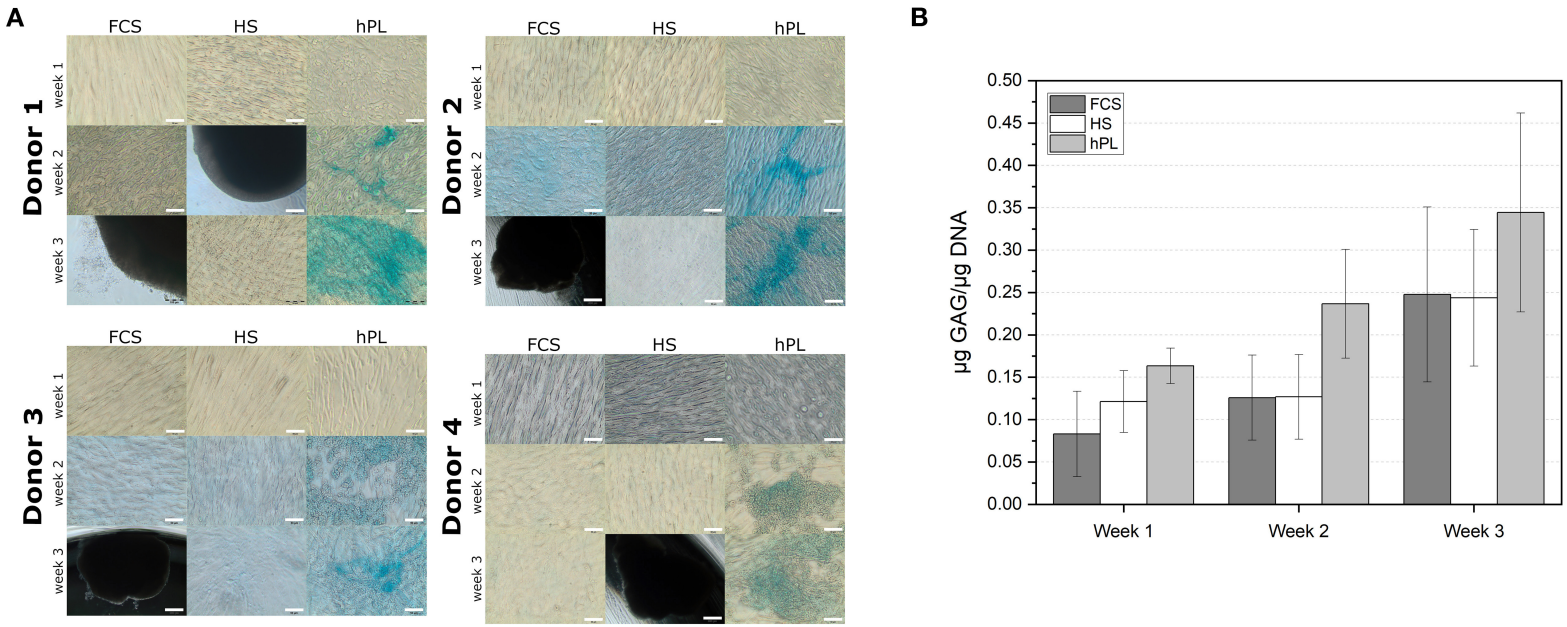
**(A)** Alcian Blue staining of the hAD-MSCs from four donors differentiated in the presence in 2.5% hPL, 10% HS, or 10% FCS. 10× objective, scale bar 100 μm. **(B)** Determination of the GAG deposition in cells (in μg GAG/μg DNA) differentiated for 7, 14, and 21 days under influence of FCS, HS, and hPL. Data represent the mean ± SD of two independent experiments in threefold determination for two donors.

Traditionally MSCs are only able to differentiate into chondrocytes when cultivated in pellet 3D cultures. In this study, hPL supported chondrogenic differentiation of hAD-MSCs also in 2D cultivations system (Figure 6). If media were supplemented with FCS or HS, hAD-MSCs of all tested donors did not show accumulation of proteoglycans after the chondrogenic stimulation (Figure 6A). Under FCS and HS cultivation conditions, cells started to detach from the cell culture surface and to agglomerate already after 2 weeks of differentiation. In the presence of hPL, cells from all tested donors started to accumulate proteoglycans already after 2 weeks of stimulation, as demonstrated by the positive Alcian Blue staining. After 2 weeks of stimulation, an increased GAG production was measured in the presence of hPL (Figure 6B). hAD-MSCs cultivated with hPL-supplemented differentiation medium showed the highest GAG/DNA ratios during the entire period of differentiation. These data demonstrate that the sole presence of hPL in chondrogenic medium can induce chondrogenic differentiation and accumulation of glycosaminoglycans by hAD-MSCs.

### Comparative Analysis of the Influence of Different Medium Supplements in 3D Cultivation

To compare the influence of different supplements on hAD-MSCs cultivated in hydrogels, both addition of supplements directly into hydrogels and addition of supplements to the media were investigated (Figure 1). To evaluate the cell growth and behavior, the morphology as well as the metabolic activity of the cells was measured (CTB).

**Figure 7:**
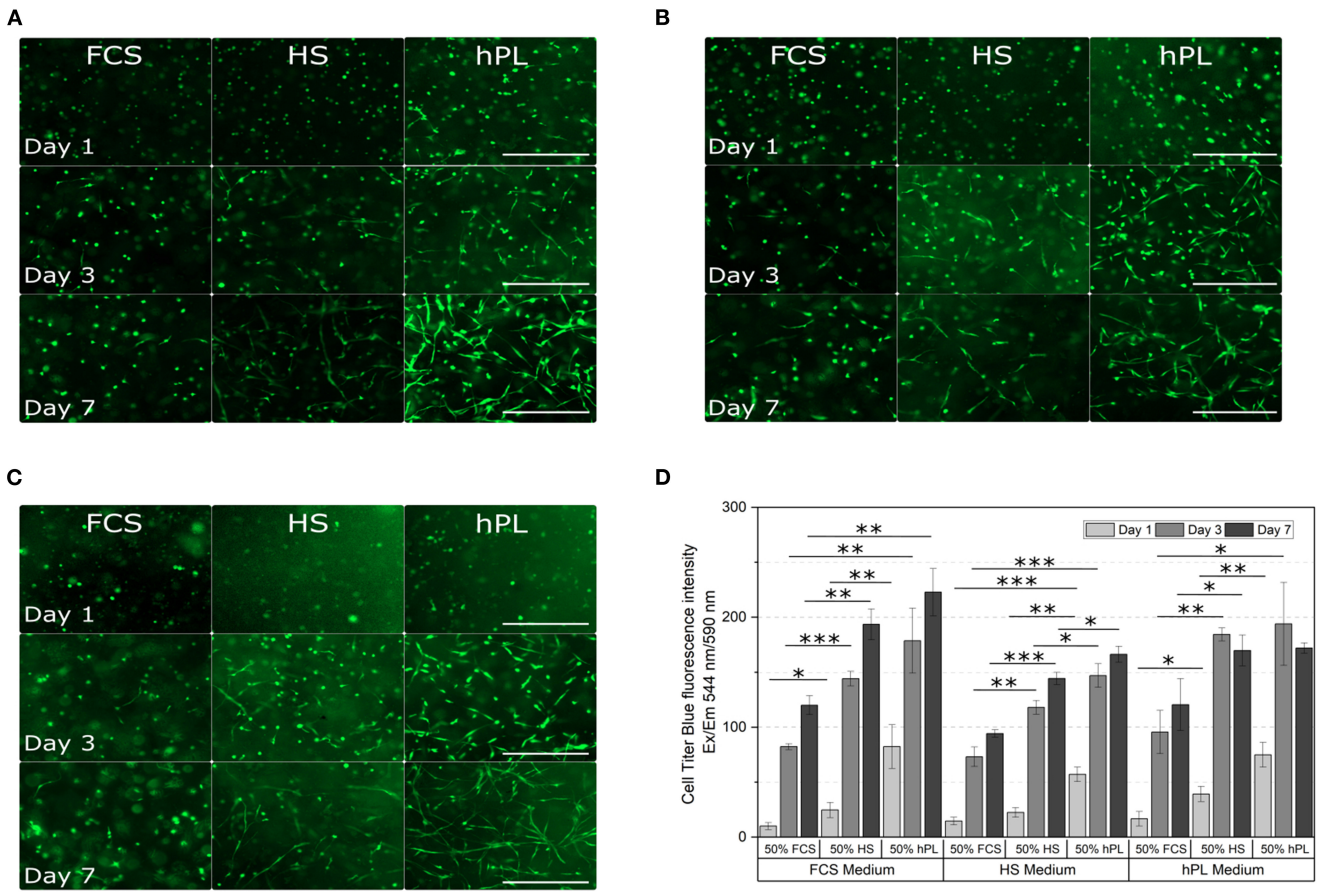
Morphological examination **(A–C)** and cell viability **(D)** of hAD-MSCs encapsulated with a UV dose of 1.2 J/cm^2^ in 5% GelMA with 50% degree of functionalization (DoF) formulated with 50% PBS and 50% FCS, 50% HS, or 50% hPL. The hydrogels were cultivated in **(A)** FCS, **(B)** HS, or **(C)** hPL supplemented medium. After cultivating the cells for 1, 3, and 7 days, they were stained with calcein-AM; 4× objective, scale bar 500 μm. **(D)** The CellTiter-Blue (CTB) assay was performed on day 1, day 3, and day 7 of cultivation. Data represent the mean ± SD for a threefold determination. *p < 0.05, **p < 0.01, ***p < 0.001.

In almost all GelMA hydrogels, increased cell spreading was visible from day three to day seven (Figure 7). The lowest amount of spreading after seven days was observed when hAD-MSCs were encapsulated in GelMA prepared in PBS with the addition of 50% FCS. When additionally cultivated with FCS-supplemented medium, spreading cells were rarely seen 7 days (Figure 7A) and only a few elongated cells were visible after 7 days in the HS or hPL-supplemented medium (Figures 7B,C). In contrast, the addition of HS to the hydrogel had a positive influence on the cell spreading in FCS, HS and hPL-supplemented medium (Figures 7A–C). Encapsulating the cells in GelMA with the addition of 50% hPL led to a more distinct cell spreading in all tested culture media. A few elongated cells could be observed already on day one with most cells showing extensive spreading after 7 days. In hPL-supplemented medium, cells were elongated and formed a three dimensional homogenous cell network.

The metabolic activity in all hydrogels increased over the cultivation time (Figure 7D). Cells encapsulated in GelMA hydrogels formulated with 50% FCS showed the lowest viability in all media, cells encapsulated in GelMA hydrogels formulated with 50% hPL demonstrated the highest viability. Formulating the hydrogels with 50% hPL instead of FCS led to an almost doubled cell viability when cultivated in FCS-supplemented medium. Regarding the media supplementation, supplementation with HS led to the lowest cell viability compared to the supplementation of FCS and hPL. However, in comparison to FCS-supplemented medium, hAD-MSCs cultivated in hPL-supplemented medium did not show a cell viability increase from day three to day seven. Overall, the cultivation of cells in GelMA hydrogels formulated with 50% FCS, in combination with HS-supplemented medium, represents the worst combination. The best combination, with the highest cell viability, was shown by GelMA hydrogels dissolved in 50% hPL, when cultivated in FCS- or hPL-containing medium.

## Discussion

Cultivation of human cells, including MSCs, in FCS-supplemented media raises concerns since cells cultivated with animal supplements can cause xenogenic immune reactions and transmit prions or other zoonotic infections after transplantation (Bottio et al., 2003; Capelli et al., 2007; Astori et al., 2016). Moreover, the large-scale manufacturing of these cells requires large amounts of clinical grade FCS, which is in limited supply. The use of serum-free media would be the best choice, however, at the moment only few chemically defined, serum-free media are available on the market (e.g., MSCGM-CD™ media from Lonza). The term “serum-free,” however, does not always mean that the media does not contain supplements of animal or human origin. Moreover, not every chemically defined medium supports cell growth of all kind of MSCs (MSCs of different origin). For example, it has been demonstrated that Mesencult-XF supports growth of hAD-MSCs, but not of bone marrow-derived mesenchymal stem cells (Al-Saqi et al., 2014). Until efficient “real” serum-free media are developed, it is essential to establish xeno-free systems for *in vitro* expansion of primary human cells, including MSCs. At the moment, more than 1,100 clinical trials are listed where MSCs from different sources are involved (clinicaltrials.gov). Treatment with MSCs often requires an *in vitro* cell expansion step to reach clinically relevant cell numbers. For each treatment, the individual cell dosage is estimated to be 10^5^-10^9^ cells (Simaria et al., 2014). In addition, the expanded MSCs should retain their biological activity (e.g., immunomodulating properties, differentiation capacity and stromal/rescue function). Thus, it is indispensable to have an efficient xeno-free *in vitro* media to expand these cells for clinical trials and later for therapies.

An additional factor which affects the cell quality is the applied cultivation system. As shown in various studies, the chosen cultivation system influences cell behavior and cell functionality, such as the expression of specific factors (Mark et al., 1977; Cukierman et al., 2001, 2002; Abbott, 2003; Bissell et al., 2003; Schmeichel and Bissell, 2003; Lee et al., 2009; Li and Cui, 2014; Papadimitropoulos et al., 2014; Sart et al., 2014; Egger et al., 2019). Therefore, the application of xeno-free *in vitro* media should be combined with cell cultivation in a 3D cultivation system, in order to obtain the best possible replication of the physiological cell state.

In the present work, the influence of hPL on proliferation and differentiation of hAD-MSCs in 2D cultivation systems was investigated and the obtained results were systematically compared with cells cultivated with HS and FCS. In this study, we revealed that the 2D supplementation of cell culture medium with 2.5% hPL accelerated cell proliferation during the first 2–3 passages. Cells isolated from all four tested donors expanded in 2D faster in the presence of 2.5% hPL than in the presence of 10% HS or 10% FCS. Although differences in proliferation capacities could be observed in each donor, the application of hPL will allow each donor to reach the fastest cell expansion. These results were demonstrated earlier for MSCs isolated from human bone-marrow, umbilical cord blood and adipose tissue (Blande et al., 2009; Shih et al., 2011; Bieback, 2013; Astori et al., 2016). It is important to note that not only supplements (serum) play an essential role in the expansion efficiency, but also the basal media composition. In addition, the right combination of basal media, supplements and seeding strategy is critical to obtain optimal conditions for *in vitro* cell growth (Kasper et al., 2018). In our work, αMEM was used, which is more physiological than the widely used high glucose DMEM or RPMI media.

Human platelets *in vivo* play a major role in homeostasis and represent a rich source of survival and growth factors, which are usually released during wound healing at the site of injury (Shih and Burnouf, 2015). Therefore, hPL could provide an efficient replacement for FCS and HS (Hemeda et al., 2014). Siciliano et al. performed the isolation and successful 2D expansion of human mediastinal hAD-MSCs in virally inactivated GMP-grade hPL (Siciliano et al., 2015). They demonstrated that mediastinal hAD-MSCs cultivated in 10–20% hPL had higher growth rates than the ones cultivated in FCS. In comparison to the above-mentioned study, we could reach the same effect by cultivating the cells with only 2.5% hPL in 2D.

In this work, we also demonstrated that hPL accelerates osteogenic and chondrogenic differentiation in 2D cultures of hAD-MSCs. Cells cultured with hPL remained viable during the entire period of differentiation. The CTB assay is regarded as an indirect measure of viability and proliferation. Since proliferation rate is significantly reduced after induction of differentiation, the low CTB values within hPL supplemented cultures indicate absence of further proliferation and initiation of osteogenic differentiation.

Furthermore, cells from all four donors accumulated calcium in the extracellular matrix already after 1 week of osteogenic stimulation. Concentrations of the extracted Alizarin red were 20 times higher than those measured after cell differentiation in HS and FCS. ALP activity could be detected after the first week, when cultured with hPL-supplemented differentiation medium. In this study, we could monitor the changes in extracellular matrix morphology during osteogenic differentiation online. By training an algorithm with the software of IncuCyte^®^ Live-Cell Imaging and Analysis, the increase of mineralization over time was detected by adding a coloring mask to the images. Using the trained mask, the software first recognized calcium deposition after seven days and evenly distributed early mineralization could be detected after only 10 days. This finding of 2D differentiated cells is in line with the results of previous studies that used hPL for 3D cell culture (Kirsch et al., 2019, 2020; Re et al., 2019). Santo et al. demonstrated that scaffolds loaded with hPL supported accelerated differentiation of hAD-MSCs (Santo et al., 2012). Another group used platelet-functionalized polycaprolactone scaffolds to enhance osteogenic differentiation of MG-63 cells (Rampichová et al., 2017). Altaie et al. reported that osteoconductive scaffolds colonized with hPL-expanded MSCs show a good capability to cure bone deficiencies *in vivo* in pre-clinical studies (Altaie et al., 2016). Since CTB was used, it is not possible to differentiate between the proliferation and metabolic activity directly. In further experiments, the cell proliferation and direct cell viability during differentiation could be measured, in order to distinguish directly between cell number and the metabolic activity of cells differentiating under the influence of the three different medium supplements.

In the case of chondrogenic differentiation in the 2D cultivation system, only cells cultivated in hPL-containing medium were positively stained with Alcian blue. The production of GAG could be already measured in the second week, if cultivated in the presence of hPL. In addition, hPL cultivated cells demonstrated the highest GAG/DNA ratios during the entire period of differentiation. Moreover, the Alcian blue staining was already positive after 2 weeks of stimulation. It is well-known that for most of the protocols for chondrogenic differentiation, cell pellets or 3D constructs need to be created in order to achieve a successful differentiation. In this work, the addition of hPL was sufficient for chondrogenesis in a 2D system. Merceron et al. were unable to detect any GAG production by hAD-MSCs cultured in 2D (Merceron et al., 2011). Only chondrogenic differentiated cells in 3D pellet cultures showed GAG synthesis and accumulation. In our study, we could show a clear Alcian blue staining, as well as a GAG production of cells differentiated with hPL in 2D. Earlier, Feng et al. demonstrated that 10% hPL could improve chondrogenic differentiation of umbilical cord-derived MSCs in 3D pellets (Feng et al., 2011). It is known that platelets contain numerous growth factors involved in chondrogenesis such as transforming growth factor-β1, vascular endothelial growth factor (VEGF), platelet-derived growth factor (PDGF), insulin-like growth factor 1 (IGF-1), and insulin-like growth factor 2 (IGF-2) (Kabiri et al., 2014). Several groups also demonstrated a positive influence of platelet-rich plasma on chondrogenesis of various MSCs in terms of increased collagen II production as well as upregulation of SOX9 and Aggrecan (Mishra et al., 2009; Xie et al., 2012; Kabiri et al., 2014). Since chondrogenic differentiation of MSCs represents a promising strategy for cartilage regeneration or replacement, application of hPL can provide good conditions for effective *in vitro* induction of chondrogenesis (Boeuf and Richter, 2010). 2D culture applications of osteogenic and chondrogenic differentiated MSCs may be of special interest for high-throughput screening. For instance, they could be used for drug screening in bone and cartilage disease models. Furthermore different studies have shown that osteogenic or chondrogenic pre-differentiated MSCs in 2D can produce cartilage and bone tissue *in vivo* in the same degree as MSCs pre-differentiated in 3D (Merceron et al., 2011).

In addition to the need for xeno-free 2D cell culture protocols, the demand for xeno-free cultivation protocols for 3D cell cultures is also increasing. In the case of hydrogel-based 3D cultivation systems, supplements can be added not only to the medium, but also directly during hydrogel formulation. Although 3D cell cultures with hPL as medium supplement have been studied, there are still certain areas that require further investigation. There are only a limited number of studies which investigated and compared the effect of different supplements on MSCs in general 3D cell culture systems and there are no studies about supplement addition directly to hydrogels (Santos et al., 2018; Kirsch et al., 2019; Re et al., 2019). In a recent study, we have demonstrated that the supplementation of GelMA hydrogels with different hPL concentrations had a positive effect on cell spreading, proliferation and differentiation, as well as on the material properties such as viscosity, storage modulus and swelling ratio (Kirsch et al., 2019).

With this work we have shown that not only the addition of the supplement to hydrogels, but also the supplement origin plays an important role in cell spreading and growth. Due to the direct addition of supplements to the hydrogels, proteins do not have to diffuse into the hydrogels from the media, and growth factors, adhesion factors and other bioactive proteins are directly accessible to encapsulated cells. In this work we could show that especially the addition of hPL to the hydrogel led to better cultivation conditions for the cells.

*In situ* formulation of hydrogels with hPL increased the cell spreading of hAD-MSCs compared to FCS- and HS-formulated hydrogels. Supporting our previous findings, hPL in hydrogels led to cell attachment and spreading after only 1 day of cultivation in 3D (Kirsch et al., 2019). In contrast, FCS and HS did not show a general positive influence on cell spreading and cell viability in hydrogels. Several studies have previously shown a hPL-dependent or hPL-supported increase of the proliferation rate and cell number of cells growing on the surface or encapsulated in hPL-containing gels (hPL gels/matrix) and hydrogels (Walenda et al., 2012; Babo et al., 2016; Fortunato et al., 2016; Egger et al., 2019; Re et al., 2019). In addition, the hPL enhanced cell spreading of cells encapsulated in hydrogels was previously observed (Fortunato et al., 2016; Santos et al., 2018; Jooybar et al., 2019). MSCs belong to the anchorage-dependent cells. Therefore, increased cell adhesion and enhanced cell spreading have a major impact on migration, proliferation and differentiation of MSCs (Lauffenburger and Horwitz, 1996; Trappmann et al., 2012; Wang et al., 2016; Yang et al., 2019). One reason for the positive effect of hPL on the cell attachment and spreading in the hydrogel could be the α-granules of platelets (Kirsch et al., 2019). The α-granules contain many different adhesion proteins, such as vitronectin, fibronectin, thrombospondin and von Willebrand factor, which are released during the platelet lysis (Sander et al., 1983; Wencel-Drake et al., 1985; Kore-Grodzicki et al., 1988; Heijnen and van der Sluijs, 2015; Burnouf et al., 2016). Another possible reason could be the different stiffness of the matrix material, which was shown to increase with higher hPL concentrations in the hydrogel (Kirsch et al., 2019). Indeed, several studies described the effect of the material stiffness from different matrices on the cell behavior of MSCs, embryonic stem cells and fibroblasts (Engler et al., 2004; Solon et al., 2007; Chowdhury et al., 2010; Park et al., 2011; Sun et al., 2018). For instance, Sun et al. demonstrated that a higher stiffness of fibronectin-coated polyacrylamide hydrogels led to greater spreading and adherence of BM-MSCs, as well as to an increased proliferation rate (Sun et al., 2018). However, the exact reason for the positive influence of hPL on cell adhesion and cell spreading in GelMA hydrogels needs to be investigated in more detail in further studies.

The most promising combinations for enhanced cell spreading and increased cell proliferation were observed by hAD-MSCs encapsulated in hydrogels formulated with 50% hPL cultivated in medium supplemented with 10% FCS or 2.5% hPL. For clinical studies and applications, however, the use of xeno-free cultivation methods is crucial. Therefore, the more suitable xeno-free 3D cultivation protocol would be the cultivation of MSCs in hydrogels formulated with 50% hPL in medium supplemented with 2.5% hPL. Since allogenic and autologous hPL can be added as a supplement to the hydrogel, but also to the medium, individualized xeno-free off-the-shelf TE constructs can be realized on the long-term using this optimized protocol. In this study, the focus of the 3D experiments was to study and compare cell behavior (morphology, cell spreading and metabolic activity) under all possible combinations of supplements added to the medium or during hydrogel formulation. In further experiments the influence of all possible supplement combinations on the differentiation of the encapsulated cells should be investigated.

The advantages of hPL compared to FCS and HS demonstrated in this work regarding the support of osteogenic and chondrogenic differentiation of MSCs in 2D *in vitro* cultivations must be investigated in further studies for 3D cultivation systems. For example, immunohistological staining of differentiation specific markers can be performed inside the hydrogel, as well as after the preparation of cryosections. Cryosections can be histochemically stained for osteogenic (e.g., calcium or alkaline phosphatase activity) and chondrogenic (e.g., collagen, proteoglycan, or glycosaminoglycan) specific markers. Moreover, the hydrogels can also be enzymatically digested in order to liberate encapsulated cells and allow the performance of cell analysis protocols like gene microarray analysis or flow cytometry.

## Conclusion

Our work demonstrated that medium supplementation with 2.5% hPL is favorable for cultivation of hAD-MSCs in 2D systems and accelerates proliferation as well as osteogenic and chondrogenic differentiation of these cells. To our knowledge, the progress of osteogenic differentiation under the influence of three different supplements was monitored and evaluated in 2D for the first time. Both, osteogenic and chondrogenic differentiation was already detectable after 1 week of stimulation. In 3D systems, we could show that hAD-MSCs in hPL-supplemented hydrogels cultivated with hPL-supplemented medium adhere and spread faster and in higher numbers when compared to FCS and HS as supplement in hydrogels or medium. This indicates that hPL can be a possible xeno-free alternative to the widely used FCS not only in 2D, but also in hydrogel-based 3D cultivation protocols. Until efficient chemically defined serum-free media are established and approved for the large-scale MSCs production and differentiation, hPL can serve as a suitable supplement for xeno-free cell cultivation in 2D and 3D. Produced under optimal conditions of standardization and safety, hPL can become a key supplement for *ex vivo* production of MSCs and *ex vivo* tissue formation for applications in the field of regenerative medicine. As yet, the precise hPL composition and the reason for fast MSCs differentiation in hPL are unclear.

Further studies including hPL fractionation, protein separation and MS-analysis must be performed in order to elucidate the positive effects of individual components of hPL and for the creation of defined supplements. Taken together, the application of hPL in 2D and 3D *in vitro* cultivation of MSCs appears to be a promising approach for bioregenerative medicine.

## Data Availability Statement

The raw data supporting the conclusions of this article will be made available by the authors, without undue reservation.

## Ethics Statement

Human AD-MSCs were isolated from adipose tissue of four donors after abdominoplasty surgery. The use of human tissue from patients (after their informed consent) has been approved by the Institutional Review Board (Hannover Medical School, Ref. Nr.: 3475-2017).

## Author Contributions

MK performed and planned the experiments and wrote the manuscript. AL and TS planned the experiments and proofread the manuscript. AS, JR, and WH prepared the hPL, participated in the experimental design and in proofreading. IP provided the GelMA and proofread the manuscript. SS and PV provided adipose tissue and cell isolation protocols and proofread the manuscript. All authors contributed to the article and approved the submitted version.

## Conflict of Interest

The authors declare that the research was conducted in the absence of any commercial or financial relationships that could be construed as a potential conflict of interest.
