# Supplementary material for: Comparative Analysis of Mesenchymal Stem Cell Cultivation in Fetal Calf Serum, Human Serum, and Platelet Lysate in 2D and 3D Systems
Source: Front Bioeng Biotechnol. 2021 Jan 15;8:598389. doi: 10.3389/fbioe.2020.598389 (PMC7844400; doi:10.3389/fbioe.2020.598389)
Supplement: Supplementary file 1 [file Table_1.docx]

Supplementary Material

**Supplementary Table 1:** Overview of the studies on the influence of hPL on proliferation and differentiation of MSCs compared to FCS.

| Heparin addition | Concentration | Proliferation | Osteogenic  differentiation | Chondrogenic Cultivation type | Chondrogenic  differentiation | References |
| --- | --- | --- | --- | --- | --- | --- |
| No | 10% hPL | hPL favorable | hPL favorable | 3D | hPL favorable | (Ben Azouna et al., 2012) |
|  |  | hPL favorable | no difference | 3D | no difference | (Mojica-Henshaw et al., 2013) |
|  |  | hPL favorable | hPL favorable | 2D | hPL favorable | (Kao et al., 2016) |
|  | 5% hPL | hPL favorable | hPL favorable | 3D | hPL favorable | (Crespo-Diaz et al., 2011), (Ben Azouna et al., 2012) |
|  | 0.75% hPL | no difference | hPL favorable | 3D | no difference | (Cowper et al., 2019) |
| Yes | 10% hPL | hPL favorable | hPL favorable | not investigated | not investigated | (Schallmoser et al., 2007), (Horn et al., 2010), (Kinzebach et al., 2013) |
|  |  | hPL favorable | no difference | not investigated | not investigated | (Bieback et al., 2009), (Blande et al., 2009), (Fernandez-Rebollo et al., 2017) |
|  |  | hPL favorable | diverse effects | not investigated | not investigated | (Cholewa et al., 2011) |
|  |  | hPL favorable | hPL favorable | 3D | hPL favorable | (Jonsdottir-Buch et al., 2013a) |
|  |  | no difference | FCS favorable | 2D | FCS favorable | (Reinisch et al., 2007) |
|  | 5% hPL | hPL favorable | no difference | 3D | no difference | (Becherucci et al., 2018), (Prins et al., 2009) |
|  |  | hPL favorable | hPL favorable | 3D | hPL favorable | (Doucet et al., 2005), (Hildner et al., 2015) |
|  |  | hPL favorable | hPL favorable | 3D | no difference | (Salvadè et al., 2010) |
|  |  | hPL favorable | no difference | 3D | hPL favorable | (Lange et al., 2007) |
|  |  | hPL favorable | no difference | not investigated | not investigated | (Chevallier et al., 2010), (Capelli et al., 2007), (Bernardo et al., 2007) |
|  |  | FCS favorable | no difference | not investigated | not investigated | (Müller et al., 2009) |

**Supplementary Table 2**: MSC specific antigen expression (CD45, CD44, CD90, CD31, CD34) of four different donors in passage 5.

| **Marker** | Donor 1 | | | Donor 2 | | |
| --- | --- | --- | --- | --- | --- | --- |
|  | **FCS** | **HS** | **PL** | **FCS** | **HS** | **PL** |
| CD45 | 0.46 % | 0.17 % | 0.01 % | 0.01 % | 0.00 % | 0.00 % |
| CD44 | 99.27 % | 96.32 % | 92.23 % | 98.03 % | 98.45 % | 96.11 % |
| CD90 | 99.55 % | 99.63 % | 99.60 % | 99.43 % | 99.96 % | 98.22 % |
| CD31 | 1.13 % | 7.83 % | 1.92 % | 0.17 % | 1.09 % | 0.13 % |
| CD34 | 0.06 % | 0.03 % | 0.01 % | 0.05 % | 0.00 % | 0.15 % |
| ntigen Expression | | | | | | |
| **Marker** | Donor 3 | | | Donor 4 | | |
|  | **FCS** | **HS** | **PL** | **FCS** | **HS** | **PL** |
| CD45 | 0.15 % | 0.00 % | 0.00 % | 0.00 % | 0.00 % | 0.00 % |
| CD44 | 99.05 % | 95.28 % | 98.60 % | 98.24 % | 96.13 % | 99.47 % |
| CD90 | 99.96 % | 99.68 % | 99.56 % | 99.66 % | 99.71 % | 99.23 % |
| CD31 | 0.39 % | 0.78 % | 1.88 % | 1.60 % | 0.17 % | 0.65 % |
| CD34 | 0.06 % | 0.78 % | 0.00 % | 0.14 % | 0.02 % | 0.11 % |


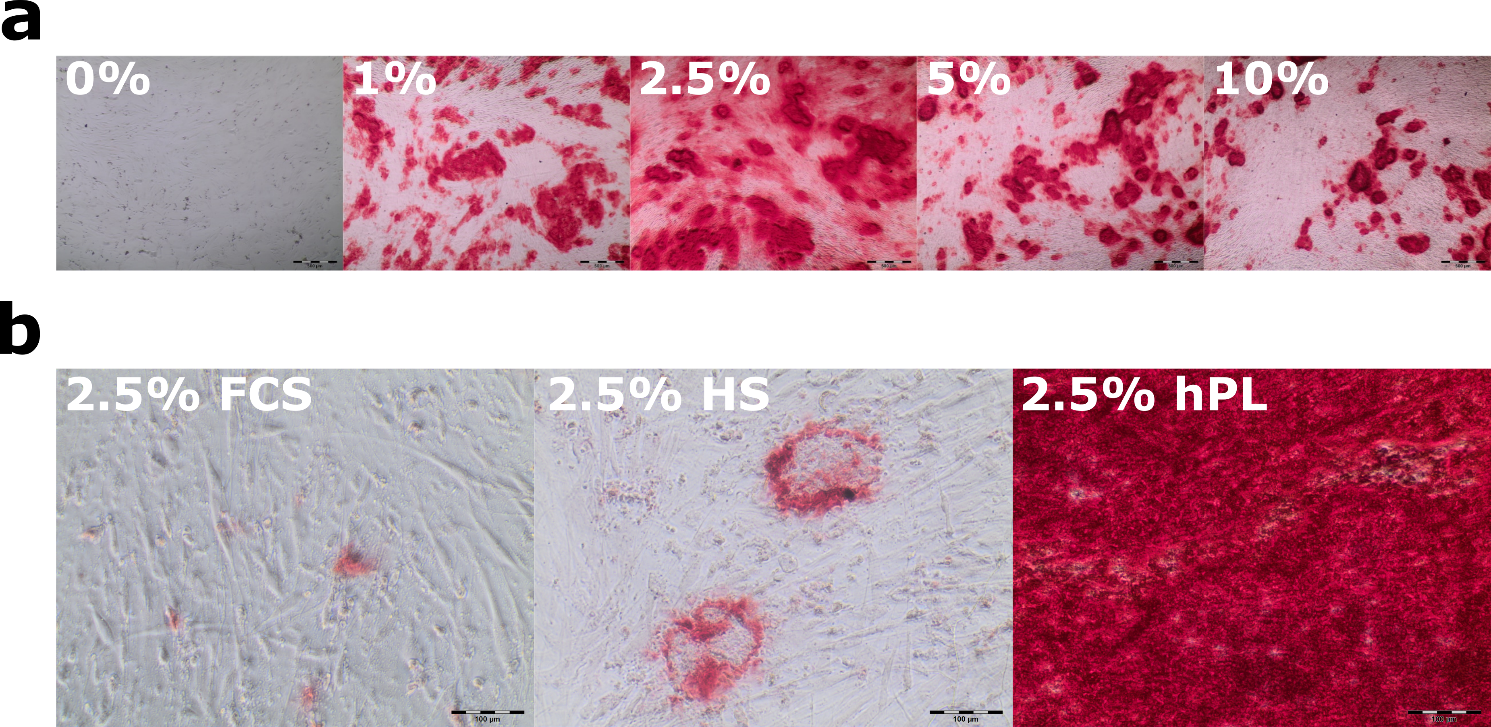


**Supplementary Figure 1.** Alizarin red staining of hAD-MSCs differentiated in the presence of (**A**) 0%, 1%, 2.5%, 5% or 10% hPL and (**B**) 2.5% FCS, HS or hPL.

**References**

Becherucci, V., Piccini, L., Casamassima, S., Bisin, S., Gori, V., Gentile, F., et al. (2018). Human platelet lysate in mesenchymal stromal cell expansion according to a GMP grade protocol: A cell factory experience. *Stem cell research & therapy* 9, 124. doi: 10.1186/s13287-018-0863-8

Ben Azouna, N., Jenhani, F., Regaya, Z., Berraeis, L., Ben Othman, T., Ducrocq, E., et al. (2012). Phenotypical and functional characteristics of mesenchymal stem cells from bone marrow: Comparison of culture using different media supplemented with human platelet lysate or fetal bovine serum. *Stem cell research & therapy* 3, 6. doi: 10.1186/scrt97

Bernardo, M. E., Avanzini, M. A., Perotti, C., Cometa, A. M., Moretta, A., Lenta, E., et al. (2007). Optimization of in vitro expansion of human multipotent mesenchymal stromal cells for cell-therapy approaches: Further insights in the search for a fetal calf serum substitute. *Journal of cellular physiology* 211, 121–130. doi: 10.1002/jcp.20911

Chevallier, N., Anagnostou, F., Zilber, S., Bodivit, G., Maurin, S., Barrault, A., et al. (2010). Osteoblastic differentiation of human mesenchymal stem cells with platelet lysate. *Biomaterials* 31, 270–278. doi: 10.1016/j.biomaterials.2009.09.043

Cholewa, D., Stiehl, T., Schellenberg, A., Bokermann, G., Joussen, S., Koch, C., et al. (2011). Expansion of adipose mesenchymal stromal cells is affected by human platelet lysate and plating density. *Cell transplantation* 20, 1409–1422. doi: 10.3727/096368910X557218

Cowper, M., Frazier, T., Wu, X., Curley, L., Ma, M. H., Mohuiddin, O. A., et al. (2019). Human Platelet Lysate as a Functional Substitute for Fetal Bovine Serum in the Culture of Human Adipose Derived Stromal/Stem Cells. *Cells* 8. doi: 10.3390/cells8070724

Crespo-Diaz, R., Behfar, A., Butler, G. W., Padley, D. J., Sarr, M. G., Bartunek, J., et al. (2011). Platelet lysate consisting of a natural repair proteome supports human mesenchymal stem cell proliferation and chromosomal stability. *Cell transplantation* 20, 797–811. doi: 10.3727/096368910X543376

Hildner, F., Eder, M. J., Hofer, K., Aberl, J., Redl, H., van Griensven, M., et al. (2015). Human platelet lysate successfully promotes proliferation and subsequent chondrogenic differentiation of adipose-derived stem cells: A comparison with articular chondrocytes. *Journal of tissue engineering and regenerative medicine* 9, 808–818. doi: 10.1002/term.1649

Horn, P., Bokermann, G., Cholewa, D., Bork, S., Walenda, T., Koch, C., et al. (2010). Impact of individual platelet lysates on isolation and growth of human mesenchymal stromal cells. *Cytotherapy* 12, 888–898. doi: 10.3109/14653249.2010.501788

Kao, Y.-C., Bailey, A., Samminger, B., Tanimoto, J., and Burnouf, T. (2016). Removal process of prion and parvovirus from human platelet lysates used as clinical-grade supplement for ex vivo cell expansion. *Cytotherapy* 18, 911–924. doi: 10.1016/j.jcyt.2016.04.002

Kinzebach, S., Dietz, L., Klüter, H., Thierse, H.-J., and Bieback, K. (2013). Functional and differential proteomic analyses to identify platelet derived factors affecting ex vivo expansion of mesenchymal stromal cells. *BMC cell biology* 14, 48. doi: 10.1186/1471-2121-14-48

Müller, A. M., Davenport, M., Verrier, S., Droeser, R., Alini, M., Bocelli-Tyndall, C., et al. (2009). Platelet lysate as a serum substitute for 2D static and 3D perfusion culture of stromal vascular fraction cells from human adipose tissue. *Tissue engineering. Part A* 15, 869–875. doi: 10.1089/ten.tea.2008.0498

Prins, H.-J., Rozemuller, H., Vonk-Griffioen, S., Verweij, V. G. M., Dhert, W. J. A., Slaper-Cortenbach, I. C. M., et al. (2009). Bone-forming capacity of mesenchymal stromal cells when cultured in the presence of human platelet lysate as substitute for fetal bovine serum. *Tissue engineering. Part A* 15, 3741–3751. doi: 10.1089/ten.TEA.2008.0666

Reinisch, A., Bartmann, C., Rohde, E., Schallmoser, K., Bjelic-Radisic, V., Lanzer, G., et al. (2007). Humanized system to propagate cord blood-derived multipotent mesenchymal stromal cells for clinical application. *Regenerative medicine* 2, 371–382. doi: 10.2217/17460751.2.4.371

Salvadè, A., Della Mina, P., Gaddi, D., Gatto, F., Villa, A., Bigoni, M., et al. (2010). Characterization of platelet lysate cultured mesenchymal stromal cells and their potential use in tissue-engineered osteogenic devices for the treatment of bone defects. *Tissue engineering. Part C, Methods* 16, 201–214. doi: 10.1089/ten.TEC.2008.0572
